# Sociodemographic Determinants of Willingness and Extent to Pay for COVID-19 Vaccine in India

**DOI:** 10.3389/fpubh.2022.870880

**Published:** 2022-06-06

**Authors:** Tanvi Kiran, K. P. Junaid, Divya Sharma, Lovely Jain, Jatina Vij, Prakasini Satapathy, Venkatesan Chakrapani, Binod Kumar Patro, Sitanshu Sekhar Kar, Ritesh Singh, Star Pala, Surya Bali, Neeti Rustagi, Kapil Goel, Lalit Sankhe, Bhavesh Modi, Madhu Gupta, Arun Kumar Aggarwal, Vineeth Rajagopal, Bijaya Kumar Padhi

**Affiliations:** ^1^Department of Community Medicine and School of Public Health, Postgraduate Institute of Medical Education and Research, Chandigarh, India; ^2^Department of Public Health, Utkal University, Bhubaneswar, India; ^3^Centre for Sexuality and Health Research and Policy, Chennai, India; ^4^Department of Community Medicine and Family Medicine, All India Institute of Medical Sciences Bhubaneswar, Bhubaneswar, India; ^5^Department of Preventive and Social Medicine, Jawaharlal Institute of Postgraduate Medical Education and Research, Puducherry, India; ^6^Department of Community Medicine and Family Medicine, All India Institute of Medical Sciences, Kalyani, India; ^7^North Eastern Indira Gandhi Regional Institute of Health and Medical Sciences, Shillong, India; ^8^Department of Community and Family Medicine, All India Institute of Medical Sciences Bhopal, Bhopal, India; ^9^Department of Community Medicine and Family Medicine, All India Institute of Medical Sciences Jodhpur, Jodhpur, India; ^10^Grant Medical College, Sir Jamshedjee Jeejeebhoy Group of Hospitals, Mumbai, India; ^11^Department of Community and Family Medicine, AIIMS-Rajkot, Gujarat, India

**Keywords:** COVID-19, vaccine, willingness to pay, sociodemographic factors, India

## Abstract

**Background:**

Responding to the fast transmission rates and increasing fatality rates, countries across the world expedited the development and deployment of the vaccine for coronavirus disease 2019 (COVID-19). Evaluation of individuals' willingness to pay (WTP) would provide pertinent information regarding future demand and financing preferences, which shall help to devise the effective payment strategy for COVID-19 vaccination.

**Methods:**

A nationwide, cross-sectional, and self-administered online survey using a structured questionnaire was conducted to identify the sociodemographic determinants of willingness and extent to pay for COVID-19 vaccine in India. A non-probability convenience sampling followed by snowball sampling was employed to recruit participants (*n* = 3,341). The likelihood of sociodemographic determinants to predict willingness and extent to pay was modeled using the multivariate binary logistic regression analysis.

**Results:**

Out of 3,341 participants, 68% (*n* = 2,271) were willingness to pay for COVID-19 vaccine. Results showed significantly higher odds for willingness to pay among participants who were single [adjusted odds ratio (aOR) = 1.394, *p* < 0.01] and having a family size of 4 members (aOR = 1.346, *p* < 0.01). The adjusted odds ratio sizably increased from 1.396 for participants whose monthly income was between INR 10,000 and 20,000/month to 2.240 for participants whose monthly income was above INR 50,000/month. Further, out of 2,271 of those participants who were willingness to pay for COVID-19 vaccine, majority (*n* = 1,246, 54.9%) of participants were willingness to pay below 50% of COVID-19 vaccine cost. This study found that those who are single (aOR = 0.688, *p* < 0.01), having an income between INR 20,000 and 50,000/month (aOR = 0.686, *p* < 0.05), and those who belonged to socially disadvantaged category (aOR = 0.450, *p* < 0.01) were estimated to have significantly lower odds of willingness to pay more than 50% of COVID-19 vaccine cost.

**Conclusion:**

This study observed that majority of those participants who willingness to pay for COVID-19 vaccine were willingness to pay only up to 50% of COVID-19 vaccine and income was observed as a precursor predictor of the willingness and extent to pay for COVID-19 vaccine. The understanding on the willingness and extent to pay for COVID-19 vaccine and its sociodemographic determinants will be helpful for making the strategic decisions related to the financing of COVID vaccine in India.

## Introduction

The unprecedented coronavirus disease 2019 (COVID-19) crisis has posed a substantial threat to global public health. COVID-19 has reported over 374.7 million cases and has claimed 5.66 million lives globally as on 31 January 2022 (1). The pandemic has resulted in a significant change in the behavior of our society and impacted individuals' physical and mental health (2). Due to the lockdown imposed worldwide to curb the spread of coronavirus disease 2019, the economic activities also suffered a setback, causing a reduction in employment opportunities (3). Coronavirus disease 2019 has impacted people's lives considerably by affecting their health and economic wellbeing (4).

As no antiviral drug has been accepted for the effective treatment of the novel coronavirus disease 2019 so far, vaccination has been identified as a crucial intervention to control its spread and potency (5). Vaccines are the most cost-effective health technology to contain an infectious disease and play a significant role in averting deaths and hospitalization caused by coronavirus disease 2019 (6). Responding to the fast transmission rates and increasing fatality rates (7), countries across the world dedicated themselves to develop the vaccine for coronavirus disease 2019. The global partnership culminated in the fastest development of a vaccine in history. Now that, the vaccine is available in the market; pharmaceutical companies, research organizations, and governments worldwide are expediting the vaccine's preparation, manufacturing, and administration to vaccinate maximum individuals across the varied age groups (8).

However, many challenges are associated with large-scale production and equitable access and distribution of the vaccine. The manufacturers and the government are accelerating their efforts to achieve maximum vaccination coverage on the supply side. Still, the public's unexplored perception or valuation for COVID-19 vaccination on the demand side may hamper the successful establishment of vaccination campaigns (8). The vaccination cost, including the vaccine expenses, its dissemination, and administration, is sizably high (8). Currently, the government across the world, including India, is bearing the maximum cost of the vaccine and is making it available for free for the population (9). But, the cost of vaccine production and other indirect costs will lead to an immense fiscal burden on the government in the future (8). With the subsequent booster doses required to control the current pandemic and its future outbreaks, strategies are required to address issues of the financial affordability of the vaccine (10). The governments worldwide cannot regularly pay for booster shots for the population, as it adds to the fiscal deficit. The government may need to modify its course of action and require the public to pay a proportion of the vaccination cost, given its budget limitations on healthcare spending and the economic burden (3).

The cost of vaccination is a significant factor associated with the extent of willingness (11) to uptake the vaccine despite its known efficacy. Therefore, it becomes imperative to know if the people would be willing to purchase the vaccine. The willingness to pay (WTP) for vaccination is a monetary measure of people's inclination and perception for vaccination, indicating the trade-off between the benefits of the vaccination and the personal economic cost (12, 13). Evaluation of individuals' WTP would provide pertinent information regarding future demand and financing preferences, which shall help to devise the best payment strategy for COVID-19 vaccination (8, 14).

Studies have found that willingness to pay is significantly affected by various sociodemographic determinants (12, 14). The relationship of these determinants with WTP varies in different communities. The sociodemographic determinants that influence WTP for vaccination include age, gender, social category, education, occupation, income, family size, marital status, etc.

The willingness to pay may be higher in older people than the younger ones, as the elderly are more vulnerable and at a relatively higher risk of getting inflicted with COVID-19 (15). In the Indian context, “Scheduled Caste” (SC), “Scheduled Tribes” (STs), and “Other Backward Classes” (OBCs) are categorized as the socially disadvantaged groups (16), as they straggle behind the others due to their economic and social backwardness. Both the gender and being part of socially disadvantaged group affect the willingness to pay for the vaccine. A study reported that females and the Schedule Caste/Tribe social groups were willingness to pay lesser for COVID-19 vaccination (5). Education can positively influence WTP, as more knowledge increases awareness of the disease. Occupation can significantly predict willingness to pay, as individuals associated with healthcare or public dealing would be more willingness to pay, considering the higher risk of infection. However, some studies have also reported that occupation is not a significant predictor for vaccination (17) and those with better educational status (having Bachelor's degree) was negatively associated with COVID-19 vaccination (18). Individuals with low intention for vaccine uptake also negatively influence willingness to pay for the vaccine cost. Income has a strong association with WTP, as individuals with higher income can afford the vaccine and would be willingness to pay more (5, 8). Family size may also significantly affect willingness to pay because an increase in family size would increase the financial burden, thereby lowering saving-income ratios (19). Similarly, an unmarried/single individual may be more willingness to pay due to less financial responsibilities than a married individual.

It is evident that the sociodemographic indicators influence WTP to a great extent. Hence, it becomes vital for the government and organizations to identify the determinants affecting WTP for COVID-19 vaccination and their association to formulate interventions for specific populations (14). Literature on WTP estimation and identification of the related determinants for COVID-19 vaccine in India is limited. In this context, this study was conducted to identify the sociodemographic determinants of willingness and extent to pay for COVID-19 vaccine in India. This study covers a wider geographic area, including all the regions of India, thus it would be helpful for the government and policymakers to devise population and region-specific strategies for COVID-19 vaccine financing.

## Methodology

### Study Design and Sample

This study following a cross-sectional study design was conducted through a nationwide self-administered online survey using a structured questionnaire between the period of October 2020 and December 2020 in India. The survey was conducted before the commencement of COVID-19 vaccination in India and included only the participants aged 18 years and above who are currently living in India. This study adapted convenience sampling method to recruit the participants. The invitation link of this study questionnaire was disseminated through the social media platforms such as WhatsApp, Facebook, Twitter, Telegram, and emails. This study considered only those participants who had access to internet-enabled devices such as desktop, laptop, smartphone, tablet, and alike. This study, therefore, included only those participants who have the ability to read though and they lack formal education. Followed by that, this study further employed snowball sampling method to recruit additional participants by requesting invited participants to share the invitation link (Google Forms) to their peers and contacts. The Google Forms contained the contact details of the investigator to facilitate the participants in this study, if they require. Subsequently, a number of study participants who required assistance in filling up the form directly contacted the investigators, which further facilitated the completion of the questionnaire. The minimum required sample size ranged between 433 and 577 based on expected proportion of 25–50% population willingness to pay for vaccine with 5% absolute precision, 1.5 as the assumed design effect, and 95% CI (20). Nevertheless, this study recruited a total of 3,341 participants to understand the factors associated with willingness to pay.

### Study Questionnaire

The questionnaire for the survey was prepared in the English language and was translated to local language for the dissemination of the questionnaire nationwide using the Google Forms. This self-administered questionnaire primarily consisted of multiple sections, such as sections assessing sociodemographic characteristics, knowledge and perception toward COVID-19, information sources of vaccine, and participant's willingness to accept and to pay for the proposed COVID-19 vaccine (full complete doses in the vaccine schedule). Out of these varied domains of this broad questionnaire, this study focused on the sociodemographic factors associated with the willingness and extent to pay for COVID-19 vaccination in India.

### Statistical Analysis

The primary outcome of this study is to determine the proportion of population spread across the varied socioeconomic and demographic groups who are willingness to pay for COVID-19 vaccine across in India and to further identify the particular sociodemographic variables that play a significant factor to determine the willingness and extent to pay for COVID-19 vaccine. The respondents were asked that what percentage of the vaccine cost they are planning to pay comfortably. Those who marked “0%” were classified as the “not willingness to pay” group and those who marked any of the other response (25% and below, 26–50%, 51–75%, and more than 75%) were classified to the “willingness to pay” group. Descriptive statistics were performed followed by bivariate analyses to assess the presence of any association between sociodemographic variables and willingness/extent to pay for COVID-19 vaccine in India. This study employed the multivariate binary logistic regression analysis to identify the sociodemographic determinants of the willingness and extent to pay in two steps. In the first step, the multivariate binary logistic regression analysis was performed on the entire sample size of 3,341 participants. In the second step, the regression was employed on 2,271 participants who were reported to be willingness to pay for COVID-19 vaccine. The extent of willingness to pay ranged between the four categories (25% and below, 26–50%, 51–75%, and more than 75%) and since the frequency count of different categories of independent variables (sociodemographic factors) were quite low, therefore, the four categories of dependent variable (extent to willingness to pay) were merged into two categories (below 50% and above 50%) and the multivariate binary logistic regression analysis was subsequently performed (21). The adjusted odds ratios with 95% CI and *p*-values (*p* < 0.05) were reported. The data were analyzed using the IBM SPSS Statistics version 25.

### Ethical Consideration

This study was approved by the Institutional Research Ethics Committee, Post Graduate Institute of Medical Education and Research (PGIMER), <city>Chandigarh </city>, India (INT/IEC/2020/SPL-795). The informed consent was obtained at the beginning of the survey and only those participants provided consent taken to the webpage of the questionnaire.

## Results

### Willingness to Pay and Sociodemographic Factors

Table 1 presents the details of sociodemographic characteristics of the participants and proportion of willingness to pay for COVID-19 vaccine. Out of 3,341 participants, 51.4% (*n* = 1,718) were females. Majority of the participants (*n* = 1,835, 54.6%) are aged between 26 and 45 years and were reported to be married (*n* = 2,022, 60.5%). A large proportion (*n* = 1,272, 38.1%) of participants were educated up to primary school level, while 29.3% (*n* = 978) of participants were lacked formal education. Around 32.7% (*n* = 1,091) of participants were educated more than primary school level. Participants who were working (government or private employees and selfemployed) recorded the highest proportion of about 58.2% (*n* = 1,944) as compared to 22.2% (*n* = 742) of participants who were not working and 19.6% (*n* = 655) who were students. A considerable number of the participants (*n* = 1,413, 42.3%) reported having monthly income “above INR 50,000,” while 16.5% (*n* = 552) of the participants was having a monthly income below INR 10,000. Looking at the family size, 41.1% (*n* = 1,372) of the participants reported a family size of “more than 4 members.” Majority of the participants (62%, *n* = 2,073) belonged to general category, whereas socially disadvantaged categories, namely, Other Backward Class (OBC), Scheduled Caste (SC), and Scheduled Tribes (STs) recorded a proportion of 20.2 (*n* = 675), 11.6 (*n* = 387), and 6.2% (*n* = 206), respectively. Of the total participants, North region of the country recorded the highest proportion of 34.8% (*n* = 1,162), whereas the South region of the country recorded the least proportion of 14.6% (*n* = 487).

**Table 1:** Description of socio-demographic factors for willingness to pay.

**Variables**	***N* (%)**
**Gender**	
Female	1,718 (51.4)
Male	1,623 (48.6)
**Age**	
25 and below	1,024 (30.6)
26–45 years	1,835 (54.9)
46 and above	482 (14.4)
**Marital status**	
Married	2,022 (60.5)
Single (Not married/divorced/widowed/-separated)	1,319 (39.5)
**Education**	
No formal education	978 (29.3)
Primary school level	1,272 (38.1)
More than primary school	1,091 (32.7)
**Occupation**	
Not working	742 (22.2)
Student	655 (19.6)
Employed (Government or private employees and self-employed)	1,944 (58.2)
**Income**	
Below INR 10,000/month	552 (16.5)
INR 10,000 to INR 20,000/month	531 (15.9)
INR 20,000 to INR 50,000/month	845 (25.3)
Above INR 50,000/month	1,413 (42.3)
**Family size**	
More than 4 members	1,372 (41.1)
4 members	1,241 (37.1)
Less than 4 members	728 (21.8)
**Social category**	
General	2,073 (62.0)
OBC	675 (20.2)
SC	387 (11.6)
ST	206 (6.2)
**Region**	
East & north east	990 (29.6)
West & central	702 (21.0)
North	1,162 (34.8)
South	487 (14.6)

The bivariate analysis between sociodemographic characteristics and willingness to pay for COVID-19 vaccine is given in Table 2. Notably, across all the sociodemographic groups, number of participants who are willingness to pay for COVID-19 vaccine is higher than that of those who are not willingness to pay for COVID-19 vaccine (Table 2). The bivariate analysis showed that sociodemographic variables such as age, marital status, education, occupation, income, family size, and social category are significantly and independently associated to willingness to pay for COVID-19 vaccine (*p*-value < 0.05). Majority of the females (*n* = 1,166) and males (*n* = 1,105) were willingness to pay for COVID-19 vaccine and females (51.3%) recorded higher percentage than males (48.7%). Those who were willingness to pay among the “26 to 45 years” age group (53.7%) were sizably greater than the other age groups. Further, willingness to pay for COVID-19 vaccine is higher for those who were married (57.7%), those who completed their primary school level education (39.3%), those who are employed (56.7%), those having income “above INR 50,000/month” (45.7%), those with family size of four members (39.5%), and those who belong to the general category (64.5%).

**Table 2:** Bivariate analysis between sociodemographic characteristics and willingness to pay.

	**Willingness to pay**			
**Variables**	**Not willing to pay (*n* = 1,070, 32%)**	**Willing to pay (*n* = 2,271, 68%)**	**Chi-square value**	***p*-value**
**Gender**			0.018	0.894
Female	552 (51.6%)	1,166 (51.3%)		
Male	518 (48.4%)	1,105 (48.7%)		
**Age**			11.673	0.003
25 and below	286 (26.7%)	738 (32.5%)		
26–45 years	616 (57.6%)	1,219 (53.7%)		
46 and above	168 (15.7%)	314 (13.8%)		
**Marital status**			23.150	<0.001
Married	711 (66.4%)	1,311 (57.7%)		
Single (Not married/divorced/widowed/separated)	359 (33.6%)	960 (42.3%)		
**Education**			29.368	<0.001
No formal education	274 (25.6%)	704 (31.0%)		
Primary school level	379 (35.4%)	893 (39.3%)		
More than primary school	417 (39.0%)	674 (29.7%)		
**Occupation**			7.917	0.019
Not working	229 (21.4%)	513 (22.6%)		
Student	184 (17.2%)	471 (20.7%)		
Employed (Government or private employees and self-employed)	657 (61.4%)	1,287 (56.7%)		
**Income**			45.095	<0.001
Below INR 10,000/month	228 (21.3%)	324 (14.3%)		
INR 10,000–20,000/month	192 (17.9%)	339 (14.9%)		
INR 20,000–50,000/month	274 (25.6%)	571 (25.1%)		
Above INR 50,000/month	376 (35.1%)	1,037 (45.7%)		
**Family size**			16.306	<0.001
More than four members	477 (44.6%)	895 (39.4%)		
Four members	345 (32.2%)	896 (39.5%)		
Less than four members	248 (23.2%)	480 (21.1%)		
**Social category**			22.754	<0.001
General	609 (56.9%)	1,464 (64.5%)		
OBC	242 (22.6%)	433 (19.1%)		
SC	155 (14.5%)	232 (10.2%)		
ST	64 (6.0%)	142 (6.3%)		
**Region**			2.267	0.519
East & north east	308 (28.8%)	682 (30.0%)		
West & central	214 (20.0%)	488 (21.5%)		
North	385 (36.0%)	777 (34.2%)		
South	163 (15.2%)	324 (14.3%)		

The multivariate binary logistic regression analysis between sociodemographic variables and willingness to pay for COVID-19 vaccine is given in Table 3. The Nagelkerke *R*^2^ showed that the model is explaining 5.8% of the variation in willingness to pay for COVID-19 vaccine. The Omnibus Tests of Model Coefficients gives a chi-square of 141.227 significant beyond 0.001 indicates that the model (with explanatory variables) has a good fit. The analysis estimated the overall accuracy of 69.7% in correctly predicting the probabilities. Sociodemographic variables such as marital status, education, occupation, income, family size, and category observed to be significantly associated with willingness to pay for COVID-19 vaccine after adjusting for other variables. The analysis showed significantly higher odds for willingness to pay among participants who were single than the married ones [adjusted odds ratio (aOR) = 1.394, 95% CI: 1.146–1.695, *p*-value < 0.01]. All the income categories (vs. monthly income <10,000 INR) were found to be significant (*p*-value < 0.05). The adjusted odds ratio sizably increased from 1.396 for participants whose monthly income was between INR 10,000 and 20,000/month to 2.240 for participants with monthly income above INR 50,000/month. This is indicative of the fact that higher the income, more the chances for willingness to pay for COVID-19 vaccine. The participants who were having a family size of four members (vs. more than four members) (aOR = 1.346, 95% CI: 1.134–1.597, *p* < 0.01) had significant higher odds for willingness to pay. The participants who belonged to the disadvantaged categories, namely, “Other Backward Class” (aOR = 0.797, 95% CI: 0.657–0.968, *p* = 0.022) and “Scheduled Caste” (aOR = 0.682, 95% CI: 0.541–0.860, *p*-value < 0.01), were observed to be less probable for willingness to pay. Similarly, participants who were educated more than primary school level (vs. participants who lacked formal education) (aOR = 0.666, 95% CI: 0.540–0.821, *p* < 0.001) and participants who were employed (vs. not working) (aOR = 0.599, 95% CI: 0.483–0.743, *p* < 0.01), as well those who were students (aOR = 0.690, 95% CI: 0.530–0.898, *p* = 0.006) registered significantly decreased odds for willingness to pay.

**Table 3:** Association between the sociodemographic variables and willingness to pay (*n* = 3,341) using the multivariate binary logistic regression analysis.

**Variable**	**Coefficient**	**Adjusted OR (95% CI)^a^**	***p*-value**
**Gender**
Female		Ref
Male	0.044	1.044 (0.896–1.218)	0.579
**Age**
25 and below		Ref
26–45 years	−0.055	0.947 (0.765–1.171)	0.613
46 and above	0.023	1.023 (0.766–1.366)	0.877
**Marital status**
Married		Ref
Single (Not married/divorced/widowed/separated)	0.332	1.394 (1.146–1.695)	0.001
**Education**
No formal education		Ref
Primary school level	−0.092	0.912 (0.751–1.107)	0.353
More than primary school	−0.406	0.666 (0.540–0.821)	<0.001
**Occupation**
Not working		Ref
Student	−0.371	0.690 (0.530–0.898)	0.006
Employed (Government or private employees and self-employed)	−0.513	0.599 (0.483–0.743)	<0.001
**Income**
Below INR 10,000/month		Ref
INR 10,000–20,000/month	0.334	1.396 (1.078–1.809)	0.011
INR 20,000–50,000/month	0.425	1.530 (1.210–1.934)	<0.001
Above INR 50,000/month	0.807	2.240 (1.767–2.840)	<0.001
**Family size**
More than four members		Ref
Four members	0.297	1.346 (1.134–1.597)	0.001
Less than four members	0.016	1.016 (0.835–1.237)	0.871
**Social category**
General		Ref
OBC	−0.226	0.797 (0.657–0.968)	0.022
SC	−0.383	0.682 (0.541–0.860)	0.001
ST	−0.061	0.941 (0.680–1.301)	0.712
**Region**
East & north east		Ref
West & central	0.050	1.051 (0.845–1.308)	0.652
North	−0.096	0.908 (0.750–1.100)	0.324
South	−0.001	0.999 (0.778–1.283)	0.994

a *Adjusted OR (95% CI) = adjusted odds ratio and the corresponding 95% confidence interval*.

*The analysis predicted probabilities for those who were willing to pay*.

*The Nagalkerke R-Square value (0.058) showed that the model explains 5.8% of the variation in willingness to pay for COVID vaccine as determined by set of socio-demographic predictors*.

*The Omnibus Tests of Model Coefficients gives a Chi-Square of 141.227 (p < 0.001) indicates the new model has a good fit*.

*The analysis estimated the overall accuracy of 69.7% in correctly predicting the probabilities*.

### Extent of Willingness to Pay and Sociodemographic Factors

Table 4 presents the bivariate analysis between sociodemographic characteristics and extent of willingness to pay for COVID-19 vaccine. Out of 2,271 participants, who were willingness to pay, around 54.9% (*n* = 1,246) were ready to pay up to 50% of COVID-19 vaccine cost and the remaining 45.1% (*n* = 1,025) were willingness to pay more than 50% of COVID-19 vaccine cost. Sociodemographic variables, namely, gender, age, marital status, education, occupation, income, family size, and social category, were found to be significantly associated with extent of willingness to pay (*p*-value < 0.05) as per the bivariate analysis. Majority of those who were willingness to pay more than 50% of COVID-19 vaccine cost are males compared to females (51.3 vs. 48.3%, *p* = 0.008). In contrast, more than half of the participants who were willing to pay up to 50% were females (53.9%) against their male counterparts (46.1%). Participants who are willingness to pay more than 50% majorly belonged to the “26 to 45 years” age group (56.2%). Similarly, married participants dominated both the group of those who “willingness to pay up to 50%” (52.2 vs. 47.8%, *p* < 0.01) and those who are “willingness to pay more than 50%” (64.4 vs. 35.6%, *p* < 0.01) of COVID-19 vaccine cost as compared to single (not married/divorced/separated/widowed) participants. Strikingly, least percentage of those participants who were willingness to pay more than 50% were educated more than primary school level as compared to participants who lacked formal education (25.1 vs. 35.7%, *p* < 0.001). A great majority of those participants who were willingness to pay more than 50% are employed as compared to non-working participants (69.4 vs. 14.3%, *p* < 0.001). The higher income groups observed to be having the higher percentage of those participants who were willingness to pay more than 50%. The participants who are in the income group of “more than INR 50,000/month” recorded to be significantly higher in number as compared to those participants who are earning INR below 1,000/month (59.8 vs. 10.3%, *p* < 0.001). Difference in the percentage distribution between the groups of those participants who are having family size less than four members (37.5%) and four members (38.4%) was minimal. Only 24.1% of those participants who willingness to pay more than 50% were having a family size more than 4 members. Around 68.4% of those participants who were willingness to pay more than 50 and 61.2% of those participants who were willingness to pay up to 50% belonged to general category.

**Table 4:** Bivariate analysis between sociodemographic characteristics and extent of willingness to pay (*n* = 2,271).

	**Extent of willingness to pay (*****n*** **=** **2,271)**			
**Variables**	**Willing to pay up to 50% (*n* = 1,246, 54.9%)**	**Willing to pay more than 50% (*n*=1,025, 45.1%)**	**Chi-square value**	***p*-value**
**Gender**			6.958	0.008
Female	671 (53.9%)	495 (48.3%)		
Male	575 (46.1%)	530 (51.7%)		
**Age**			18.112	<0.001
25 and below	450 (36.1%)	288 (28.1%)		
26–45 years	643 (51.6%)	576 (56.2%)		
46 and above	153 (12.3%)	161 (15.7%)		
**Marital status**			37.764	<0.001
Married	651 (52.2%)	660 (64.4%)		
Single (Not married/divorced/widowed/separated)	595 (47.8%)	365 (35.6%)		
**Education**			26.713	<0.001
No formal education	338 (27.1%)	366 (35.7%)		
Primary school level	491 (39.4%)	402 (39.2%)		
More than primary school	417 (33.5%)	257 (25.1%)		
**Occupation**			127.200	<0.001
Not working	366 (29.4%)	147 (14.3%)		
Student	304 (24.4%)	167 (16.3%)		
Employed (Government or private employees and self-employed)	576 (46.2%)	711 (69.4%)		
**Income**			159.864	<0.001
Below INR 10,000/month	218 (17.5%)	106 (10.3%)		
INR 10,000–20,000/month	203 (16.3%)	136 (13.3%)		
INR 20,000–50,000/month	401 (32.2%)	170 (16.6%)		
Above INR 50,000/month	424 (34.0%)	613 (59.8%)		
**Family size**			10.036	0.007
More than four members	511 (41.0%)	384 (37.5%)		
Four members	502 (40.3%)	394 (38.4%)		
Less than four members	233 (18.7%)	247 (24.1%)		
**Social category**			29.506	<0.001
General	763 (61.2%)	701 (68.4%)		
OBC	242 (19.4%)	191 (18.6%)		
SC	165 (13.2%)	67 (6.5%)		
ST	76 (6.1%)	66 (6.4%)		
**Regions**			2.195	0.533
East & north east	387 (31.1%)	295 (28.8%)		
West & central	266 (21.3%)	222 (21.7%)		
North	425 (34.1%)	352 (34.3%)		
South	168 (13.5%)	156 (15.2%)		

The results of the multivariate binary logistic regression analysis between sociodemographic variables and extent of willingness to pay for COVID-19 vaccine are given in Table 5. The analysis estimated adjusted odds ratios (aOR) for those participants who were willingness to pay more than 50% of COVID-19 vaccine cost. The Omnibus Tests of Model Coefficients recorded a chi-square of 262.487 significant beyond 0.001 that indicates that the model (with explanatory variables) has a good fit. The Nagelkerke *R*^2^ shows that the model is explaining 14.6% of the results. The analysis estimated the overall accuracy of 64.8% in correctly predicting the probability. Sociodemographic variables such as marital status, occupation, income, family size, and social category were observed to be significantly associated with extent of willingness to pay for COVID-19 vaccine after adjusting for other variables (Table 5).

**Table 5:** Association between the sociodemographic variables and extent of willingness to pay (*n* = 2,271) using the multivariate binary logistic regression analysis.

**Variable**	**Coefficient**	**Adjusted OR (95% CI)^a^**	***p*-value**
**Gender**			
Female		Ref	
Male	0.048	1.049 (0.878–1.258)	0.609
**Age**			
25 and below		Ref	
26–45 years	−0.199	0.819 (0.647–1.037)	0.098
46 and above	−0.169	0.844 (0.602–1.185)	0.328
**Marital Status**			
Married		Ref	
Single	−0.374	0.688 (0.552–0.858)	0.001
**Education**			
No formal education		Ref	
Primary school level	−0.069	0.933 (0.751–1.160)	0.535
More than primary school	−0.214	0.807 (0.626–1.040)	0.097
**Occupation**			
Not working		Ref	
Student	0.026	1.026 (0.745–1.414)	0.875
Employed (Government or private employees and self-employed)	0.658	1.930 (1.469–2.536)	<0.001
**Income**			
Below INR 10,000/month		Ref	
INR 10,000–20,000/month	0.044	1.045 (0.743–1.470)	0.800
INR 20,000–50,000/month	−0.377	0.686 (0.501–0.939)	0.019
Above INR 50,000/month	0.616	1.851 (1.361–2.516)	<0.001
**Family size**			
More than four members		Ref	
Four members	0.143	1.154 (0.944–1.412)	0.163
Less than four members	0.379	1.461 (1.148–1.859)	0.002
**Social category**			
General		Ref	
OBC	−0.144	0.866 (0.685–1.094)	0.227
SC	−0.798	0.450 (0.327–0.619)	<0.001
ST	−0.023	0.978 (0.673–1.419)	0.905
**Regions**			
East & north east		Ref	
West & central	−0.027	0.973 (0.755–1.254)	0.834
North	0.019	1.020 (0.813–1.279)	0.867
South	−0.137	0.872 (0.647–1.175)	0.367

a *Adjusted OR (95% CI) = adjusted odds ratio and the corresponding 95% confidence interval*.

*The analysis predicted probabilities for those who were willing to pay more than 50%*.

*The Omnibus Tests of Model Coefficients gives a Chi-Square of 141.227 (p < 0.001) indicates the new model has a good fit*.

*The Nagalkerke R^2^ value (0.146) showed that the model explains 14.6% of the variation in extent of willingness to pay for COVID vaccine as determined by set of socio-demographic predictors*.

*The analysis estimated the overall accuracy of 64.8% in correctly predicting the probabilities*.

This study found that those participants who are employed (vs. not working) (aOR = 1.930, 95% CI: 1.469–2.536, *p* < 0.001), having income above INR 50,000/month (vs. income below INR 10,000/month) (aOR = 1.851, 95% CI: 1.361–2.516, *p* < 0.001), and those participants having family size less than four members (vs. family more than four members) (aOR = 1.461, 95% CI: 1.148–1.859, *p* = 0.002) have significantly higher odds of willingness to pay more than 50% of COVID-19 vaccine cost. In contrast, the variable groups such as those participants who are single (not married/divorced/separated/widowed) (vs. married) (aOR = 0.688, 95% CI: 0.552–0.858, *p* = 0.001), having an income between INR 20,000 and 50,000/month (vs. income below INR 10,000/month) (aOR = 0.686, 95% CI: 0.501–0.939, *p* = 0.019), and those who belonged to the SC category (vs. general social category) (aOR = 0.450, 95% CI: 0.327–0.619, *p* < 0.001) were estimated to have significantly lower odds of willingness to pay more than 50% of COVID-19 vaccine cost.

## Discussion

Studies conducted in low- and middle-income countries (LMICs) such as Malaysia (22), Indonesia (14), and Ecuador (23) reported that majority of the participants were willingness to pay for COVID-19 vaccine. Similarly, this study also recorded that around 68% of the participants were willingness to pay for COVID-19 vaccine, thereby indicating relatively high demand for COVID-19 vaccine. This can be attributed to the high impact of COVID-19 on health and economic life of the people (24). Literature on willingness to pay for vaccine in India is limited. One such study conducted in Bhopal district of India observed that despite high acceptance of COVID-19 vaccine among people, willingness to pay for COVID-19 vaccine is minimal (5), which is in contrast with this study. The difference may be due to the study design and methods adopted. Moreover, this particular study was conducted only in a particular geographical location in India. This study, in fact, is unique in itself, as it is one of the initial attempts made to assess the willingness and extent to pay for the proposed vaccine and to further examine its association with sociodemographic factors through a large online survey spanning across major geographical regions of India.

Results of the multivariate binary logistic regression analysis observed that marital status, education, occupation, income, family size, and social category are significant predictors of willingness to pay for COVID-19 vaccine. Specifically, being single, belonging to the higher-income group, and having a less family size have significantly higher odds for willingness to pay for COVID-19 vaccine. In the previous study conducted in India, those who were single recorded higher percentage in intention to receive vaccine than those who were married (25), which indicates high demand for COVID-19 vaccine among this group. In this study, respondents having monthly income measured between INR 10,000 and 20,000, INR 20,000 and 50,000, and more than INR 50,000 recorded higher odds for willingness to pay. Most of the studies related to willingness to pay for COVID-19 vaccine, observed increasing income as a precursor predictor for willingness to pay (5, 8, 14, 22, 24). Hence, these study findings on income are in line with the existing literature. The participants of those who are having a family size of 4 members and less than four members recorded higher odds of 1.346 and 1.016, respectively, for willingness to pay. It indicates that the increased family size acts as an affordability barrier for the members to pay for COVID-19 vaccine cost. Literary evidence has also revealed that absence of affordability barrier is positively associated to the willingness to pay for COVID-19 vaccine (22). Similarly, the disadvantaged social categories such as OBC and SC registered lower odds than general category for willingness to pay.

Strikingly, this study found that those who were educated more than primary school level, which includes even those who completed graduation and postgraduation level recorded less odds for willingness to pay than those who lacked formal education, which is inconsistent with the existing literature in India (5). Similarly, this study found lower odds willingness to pay among participants who are employed than those who are not working. A study conducted in Malaysia, a LMIC, observed that education and employment level are not a significant predictor of willingness to pay for COVID-19 vaccine (11). In contrast, another study conducted in Ecuador observed that employment status is a significant positive predictor, whereas education level is insignificant predictor of willingness to pay (23). Many other studies in LMICs did not report education and occupation level, a significant predictor of willingness to pay for COVID-19 vaccine, though they found related variables such as being healthcare worker, knowledge toward disease and vaccination, employee size in workplace, and others significantly associated with willingness to pay (8, 14, 22). A study in Chile described that a great majority opined that government should finance the vaccine for all (24). Similarly, in another study, 63.92% discoursed that the government should provide COVID-19 vaccine for free (23). A study conducted in China reported that large proportion of the respondents believe that government and health insurance should pay finance some or all of COVID-19 vaccine cost (8). This could be the prime reason behind the educated and those who are employed were not willingness to pay for COVID-19 vaccine, as they may believe that it is the government or employers' responsibility to provide COVID-19 vaccines for its citizen and employees, respectively.

This study further explored the sociodemographic determinants of extent of willingness to pay. It recorded 54.9% of those participants who were willingness to pay for COVID-19 vaccine that were ready to pay up to 50% of COVID-19 vaccine cost. Previous study conducted in India reported an average willingness to pay around INR 141 (USD 1.9) for COVID-19 vaccine with full efficacy (5). In Malaysia, around 28.9% of the respondents willingness to pay an amount of USD 23 (22), whereas in Indonesia, a majority of 78.3% were willingness to pay USD 57.2 for COVID-19 vaccine (14)In this study, the multivariate binary logistic regression analysis results for extent of willingness to pay indicated that those who are employed, having higher income, and family size of less than four members have significantly higher odds of willingness to pay more than 50% of COVID-19 vaccine cost. Further, this study found those who are single, having an income between INR 20,000 and 50,000/month, which represents the middle-income class in India (26), and those who belonged to SC category registered significantly lower odds for willingness to pay more than 50% of COVID-19 vaccine cost. Notably, specific sociodemographic determinants such as having above INR 50,000 per month and having family size less than four members found to be positively predicting both the willingness and extent to pay for COVID-19 vaccine, while factor namely belonging to a disadvantaged category negatively predicts the willingness and extent to pay for vaccine.

This study faces some limitations. First, as the study followed non-probability-based convenient and snowball sampling method to collect the required information, there may be a possible selection bias as the sample was not chosen at random. Further, this study cautions possible information bias, since the respondents may have a tendency to give socially-desirable responses. Therefore, the generalization of the findings of this study needs to be made with caution. Second, since this study followed cross-sectional design, the sociodemographic predictors of willingness and extent to pay for COVID-19 vaccine point toward associational relationships and not necessary a causal one. Longitudinal studies need to be conducted especially during different phases of the pandemic to confirm the causal temporal relationships with willingness and extent to pay for COVID-19 vaccine. Third, though the study attempted to cover the respondents from all the geographical locations of India, however, the southern Indian region elicited relatively less respondents and, therefore, was less represented as compared to other regions. Fourth, a sizable portion of the sample (around 32%) was not willingness to pay for the vaccine; the reasons for this need to be further explored possibly through mixed method approaches such as in-depth interviews, focus group discussions, and alike.

Despite the limitations, it is worth mentioning that the concept of willingness and extent to pay for COVID-19 vaccine and its sociodemographic determinants are not much discussed topics in India. The sample covered by this study through a nationwide online survey is relatively large (3,341 participants) comparing to previous nationwide studies on the similar theme (11, 22). Further, this study has provided a crucial information on the proportion of people who were willingness to pay COVID-19 vaccine cost up to certain magnitude, which can be used as a starting point for conducting similar temporal studies on the same theme to gather more evidence to identify a plausible range within which majority of Indians are willingness to pay out for COVID-19 vaccination out of their own pocket. In addition, this study attempted to explore and identify the specific sociodemographic factors that significantly predict both the willingness and extent to pay for COVID-19 vaccine, which shall help government and policymakers to design appropriate financing strategies to fund the cost of COVID-19 vaccine.

## Conclusion

The devastating impact of COVID-19 on physical, mental, social, and economic life of the people has increased the demand for COVID-19 vaccine. This study is helpful for making the strategic decisions related to the financing of COVID-19 vaccine in India. This study observed that majority of those participants who willingness to pay for vaccine were willingness to pay only up to 50% of COVID-19 vaccine and income was observed as a precursor predictor of the willingness and extent to pay for vaccine. The honorable Prime Minister of India in his monthly radio broadcast announced booster dose for health and frontline workers (27), possibly extending to the public as well. India is a country with mixed health system where the involvement of private players in the vaccination is imperative to meet the increased demand for COVID-19 vaccine. Hence, the understanding on the willingness and extent to pay for COVID-19 vaccine and its sociodemographic determinants will help in effective and optimal financing and deployment strategies for COVID-19 vaccine.

## Data Availability Statement

The raw data supporting the conclusions of this article will be made available by the authors, without undue reservation.

## Ethics Statement

The studies involving human participants were reviewed and approved by Institutional Research Ethics Committee (INT/IEC/2020/SPL-795), Post Graduate Institute of Medical Education and Research (PGIMER), Chandigarh, India. The patients/participants provided their written informed consent to participate in this study.

## Author Contributions

TK and BPad conceptualized the study and designed the tools. TK, BPad, KJ, DS, LJ, JV, PS, VC, BPat, SK, RS, SP, SB, NR, VR, KG, BM, LS, MG, and AA conducted the study at national level and collected the data. All authors reviewed drafts, provided edits, and approved the final submission of the manuscript.

## Conflict of Interest

The authors declare that the research was conducted in the absence of any commercial or financial relationships that could be construed as a potential conflict of interest.

## Publisher's Note

All claims expressed in this article are solely those of the authors and do not necessarily represent those of their affiliated organizations, or those of the publisher, the editors and the reviewers. Any product that may be evaluated in this article, or claim that may be made by its manufacturer, is not guaranteed or endorsed by the publisher.
