# Erratum: Sociodemographic determinants of willingness and extent to pay for COVID-19 vaccine in India

**DOI:** 10.3389/fpubh.2022.1124119

**Published:** 2023-01-04

**Authors:** Frontiers Production Office

**Affiliations:** Frontiers Media SA, Lausanne, Switzerland

**Keywords:** COVID-19, vaccine, willingness to pay, sociodemographic factors, India

Due to a production error, there was a mistake in the formatting of Table 1 as published. The corrected Table 1 appears below.

**Table 1:** Description of socio-demographic factors for willingness to pay.

**Variables**	***N* (%)**
**Gender**	
Female	1,718 (51.4)
Male	1,623 (48.6)
**Age**	
25 and below	1,024 (30.6)
26–45 years	1,835 (54.9)
46 and above	482 (14.4)
**Marital status**	
Married	2,022 (60.5)
Single (Not married/divorced/widowed/-separated)	1,319 (39.5)
**Education**	
No formal education	978 (29.3)
Primary school level	1,272 (38.1)
More than primary school	1,091 (32.7)
**Occupation**	
Not working	742 (22.2)
Student	655 (19.6)
Employed (Government or private employees and self-employed)	1,944 (58.2)
**Income**	
Below INR 10,000/month	552 (16.5)
INR 10,000 to INR 20,000/month	531 (15.9)
INR 20,000 to INR 50,000/month	845 (25.3)
Above INR 50,000/month	1,413 (42.3)
**Family size**	
More than 4 members	1,372 (41.1)
4 members	1,241 (37.1)
Less than 4 members	728 (21.8)
**Social category**	
General	2,073 (62.0)
OBC	675 (20.2)
SC	387 (11.6)
ST	206 (6.2)
**Region**	
East & north east	990 (29.6)
West & central	702 (21.0)
North	1,162 (34.8)
South	487 (14.6)

Due to a production error, the total number of participants in the study was erroneously reported as 3,371 on two occasions.

Corrections have been made to the **Abstract**, “*Results*” subsection, paragraph 3, and to the **Results** section, “*Willingness to pay and sociodemographic factors*” subsection, paragraph 16, in which this figure was corrected to 3,341 as follows:

**Results:** Out of 3,341 participants, 68% (*n* = 2,271) were willingness to pay for COVID-19 vaccine. Results showed significantly higher odds for willingness to pay among participants who were single [adjusted odds ratio (aOR) = 1.394, *p* < 0.01] and having a family size of 4 members (aOR = 1.346, *p* < 0.01). The adjusted odds ratio sizably increased from 1.396 for participants whose monthly income was between INR 10,000 and 20,000/month to 2.240 for participants whose monthly income was above INR 50,000/month. Further, out of 2,271 of those participants who were willingness to pay for COVID-19 vaccine, majority (*n* = 1,246, 54.9%) of participants were willingness to pay below 50% of COVID-19 vaccine cost. This study found that those who are single (aOR = 0.688, *p* < 0.01), having an income between INR 20,000 and 50,000/month (aOR = 0.686, *p* < 0.05), and those who belonged to socially disadvantaged category (aOR = 0.450, *p* < 0.01) were estimated to have significantly lower odds of willingness to pay more than 50% of COVID-19 vaccine cost.

## Results

### Willingness to pay and sociodemographic factors

Table 1 presents the details of sociodemographic characteristics of the participants and proportion of willingness to pay for COVID-19 vaccine. Out of 3,341 participants, 51.4% (*n* = 1,718) were females. Majority of the participants (*n* = 1,835, 54.6%) are aged between 26 and 45 years and were reported to be married (*n* = 2,022, 60.5%). A large proportion (*n* = 1,272, 38.1%) of participants were educated up to primary school level, while 29.3% (*n* = 978) of participants were lacked formal education. Around 32.7% (*n* = 1,091) of participants were educated more than primary school level. Participants who were working (government or private employees and selfemployed) recorded the highest proportion of about 58.2% (*n* = 1,944) as compared to 22.2% (*n* = 742) of participants who were not working and 19.6% (*n* = 655) who were students. A considerable number of the participants (*n* = 1,413, 42.3%) reported having monthly income “above INR 50,000,” while 16.5% (*n* = 552) of the participants was having a monthly income below INR 10,000. Looking at the family size, 41.1% (*n* = 1,372) of the participants reported a family size of “more than 4 members.” Majority of the participants (62%, *n* = 2,073) belonged to general category, whereas socially disadvantaged categories, namely, Other Backward Class (OBC), Scheduled Caste (SC), and Scheduled Tribes (STs) recorded a proportion of 20.2 (*n* = 675), 11.6 (*n* = 387), and 6.2% (*n* = 206), respectively. Of the total participants, North region of the country recorded the highest proportion of 34.8% (*n* = 1,162), whereas the South region of the country recorded the least proportion of 14.6% (*n* = 487).

The publisher apologizes for this mistake. The original version of this article has been updated.

